# Safety and Efficacy of Percutaneous Mitral Valve Repair Using the MitraClip^®^ System in Patients with Diabetes Mellitus

**DOI:** 10.1371/journal.pone.0111178

**Published:** 2014-11-06

**Authors:** Katharina Hellhammer, Tobias Zeus, Jan Balzer, Silke van Hall, Christos Rammos, Rabea Wagstaff, Malte Kelm, Tienush Rassaf

**Affiliations:** University Hospital Düsseldorf, Medical Faculty, Dept. of Medicine, Division of Cardiology, Pulmonology and Vascular Medicine, Düsseldorf, Germany; KRH Robert Koch Klinikum Gehrden, Germany

## Abstract

**Background:**

Patients with diabetes mellitus show a negative outcome in percutaneous coronary intervention, aortic valve replacement and cardiac surgery. The impact of diabetes on patients undergoing treatment of severe mitral regurgitation (MR) using the MitraClip system is not known. We therefore sought to assess whether percutaneous mitral valve repair with the MitraClip system is safe and effective in patients with diabetes mellitus.

**Methods and Results:**

We included 58 patients with severe and moderate-to-severe MR in an open-label observational single-center study. Ninteen patients were under oral medication or insulin therapy for type II diabetes mellitus. MitraClip devices were successfully implanted in all patients with diabetes and in 97.4% (n = 38) of patients without diabetes (p = 0.672). Periprocedural major cardiac adverse and cerebrovascular events (MACCE) occurred in 5.1% (n = 2) of patients without diabetes whereas patients with diabetes did not show any MACCE (p = 0.448). 30-day mortality was 1.7% (n = 1) with no case of death in the diabetes group. Short-term follow up of three months showed a significant improvement of NYHA class and quality of life evaluated by the Minnesota Living with Heart Failure Questionnaire in both groups, with no changes in the 6-minute walk test.

**Conclusions:**

Mitral valve repair with the MitraClip system is safe and effective in patients with type II diabetes mellitus.

**Trial Registration:**

MitraClip Registry NCT02033811

## Introduction

Mitral valve regurgitation (MR) is the second common valvular heart disease and severely affects morbidity and mortality [1]–[5]. Surgical treatment for many years used to be the first line treatment for patients with severe symptomatic MR [6]. Percutaneous mitral valve repair (PMVR) with the MitraClip system, however, has emerged to an effective therapeutic alternative for patients who can not undergo surgery due to high surgical risk [7]. Many of the high-risk patients present with diabetes mellitus, which is known to worsen outcome in patients referred to percutaneous coronary intervention (PCI) [8]–[12], cardiac surgery [13]–[15] or transcatheter aortic valve replacement (TAVI) [16], [17]. The impact of diabetes on treatment of MR using the MitraClip system is not known. We therefore sought to assess whether percutaneous mitral valve repair with the MitraClip system is safe and effective in patients with diabetes mellitus.

## Methods

The protocol for this trial and supporting CONSORT checklist are available as supporting information; see Checklist S1 and Protocol S1.

### Patient selection and study design

**Figure 1:**
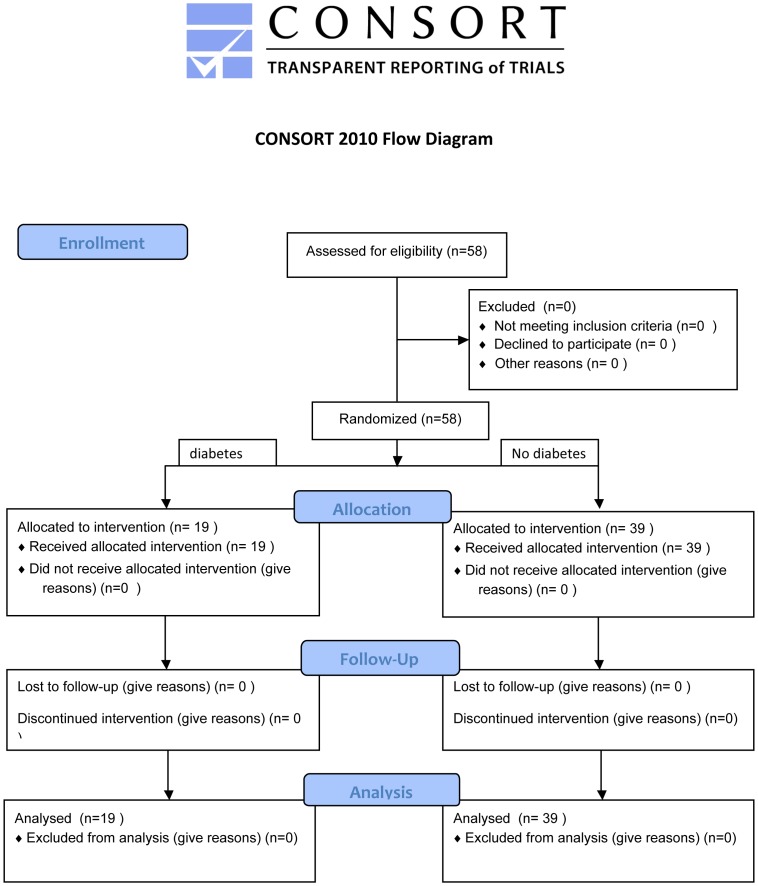
CONSORT flow chart.

A total of 58 Patients with symptomatic severe or moderate-to-severe MR evaluated by trans-thoracic and trans-esophageal echocardiography who underwent PMVR with the MitraClip System at the Heart Center Duesseldorf were included in the study (Figure 1). All patients were discussed in the institutional heart team and declined for surgical treatment due to high operative risk. Patients were included into our registry after informed written consent was obtained. The registry is registered at clinical trials (NCT02033811). In a sub-analysis of this registry, patients were grouped according to the presence of type II diabetes mellitus (oral medication or on insulin therapy). The objective of the study was to evaluate the safety and efficacy of the MitraClip procedure in patients with diabetes mellitus with a follow-up after 3 months. Study procedures were in accordance with the Declaration of Helsinki and the institutional Ethics Committee of the Heinrich-Heine University approved the study protocol.

### Data collection and definitions

Collected data included patient characteristics, imaging findings, periprocedural in hospital data, laboratory results and follow up data up to three month after PMVR. Blood samples for biochemistry and hematology analysis results were taken before the procedure. Clinical outcome parameters for follow up after three months were NYHA classification, 6-minute walk test (6MWT), and quality of life measured by the Minnesota Living With Heart Failure Questionnaire (MLHF Q). Trans-thoracic echocardiography (TTE) was performed before the procedure and during follow up. Trans-esophageal echocardiography (TEE) was performed at baseline. Severity of mitral regurgitation was classified by TTE and TEE by measuring vena contracta (VC) and regurgitation volume. A severe mitral regurgitation was defined as a VC ≥0.7 cm and a regurgitation volume ≥60 ml [18], [19].

In-hospital complications were reported for both groups. The definition of MACCE (major adverse cardiac and cerebrovascular event) included death, ST-elevation myocardial infarction, stroke and procedure related re-operation. A significant mitral stenosis after procedure was defined as a mean gradient >10 mmHg. Peripheral vascular complications were defined as minor vascular complication according to VARC II [38]. A major vascular complication was defined according to VARC II as overt bleeding either associated with a drop in hemoglobin level of at least 3.0 g/dL or requiring transfusion of two or three units of whole blood, or causing hospitalization, or permanent injury, or requiring surgery and does not meet criteria of life-threatening or disabling bleeding.

### Procedure

The MitraClip procedure was performed either in general anesthesia or deep sedation using TEE and fluoroscopy for guidance. The procedure and the MitraClip System have previously been described in detail [20], [21]. After a femoral venous access and transseptal puncture, the system is positioned in the left atrium. The arms of the clip are aligned perpendicular to the long axis of the leaflet edges and the clip is advanced into the left ventricle. After grasping the leaflets and checking the leaflet insertion the clip is deployed and the system removed.

### Statistical Analysis

Continuous data were expressed as mean ± standard deviation (SD) and compared with the unpaired student's t-test or Mann-Whitney U test if not normally distributed. Normality was checked with the Kolmogorov-Smirnov test. Categorical variables were evaluated as percentage and compared with the χ-square test or Fisher's exact test. Statistical analysis was performed with SPSS Statistics 22 (IBM) and Prism (GraphPad). A p-value <0.05 was considered to be significant.

## Results

We enrolled 58 patients with symptomatic severe and moderate-to-severe MR. Nineteen patients (32.8%) presented with diabetes mellitus type II with either oral medication or insulin therapy. Groups did not differ except for HbA1c and blood glucose levels. Baseline characteristics are summarized in Table 1.

**Table 1:** Baseline characteristics of patients undergoing MitraClip grouped according to the presence of diabetes mellitus type II.

	diabetes (n = 19)	no diabetes (n = 39)	p-value
Age, years ± SD	68±9.3	73±11.3	0.071
Male gender, n (%)	16 (84.2)	23 (59.0)	0.055
BMI, (kg/m^2^) ± SD	28±4.5	26±4.6	0.175
Logistic EuroSCORE ± SD	18.7±15.5	18.6±17.6	0.975
CAD, n (%)	11 (57.9)	24 (61.5)	0.790
COPD, n (%)	5 (26.3)	9 (23.1)	0.787
Pulmonary hypertension, n (%)	11 (57.9)	26 (66.7)	0.514
Atrial fibrillation, n (%)	5 (26.3)	15 (38.5)	0.362
Hypertension, n (%)	19 (100)	36 (92.3)	0.214
Previous CABG, n (%)	5 (26.3)	11 (28.2)	0.880
Previous valve replacement, n (%)	2 (10.5)	8 (20.5)	0.472
Previous PCI (<3 month), n (%)	1 (5.3)	1 (2.6)	0.557
Previous myocardial infarction, n (5)	9 (47.4)	13 (33.3)	0.301
NYHA class III or IV, n (%)	17 (89.5)	35 (89.7)	0.649
Chronic kidney disease, n (%)	11 (57.9)	21 (53.8)	0.771
HbA1c, % ± SD	7.9±1.4	5.9±0.5	<0.01
Glucose, mg/dL ± SD	183±54	110±26	<0.01
GFR, mL/min ± SD	51±21	61±23	0.117
BNP, pg/mL ± SD	3315±4184	2918±2805	0.686
EF, % ± SD	37±13	45±16	0.062
LVEDD, mm ± SD	61±14	59±11	0.584
MR grade III, n (%)	18 (94.7)	30 (76.9)	0.142
MR type			
functional, n (%)	17 (89.5)	36 (92.3)	0.533
degenerative, n (%)	2 (10.5)	3 (7.7)	0.533
6-MWT, m ± SD	275±90	296±121	0.511
MLHF Q ± SD	44.7±14.9	43.4±12.3	0.734

SD = standard deviation; BMI = body mass index; CAD = coronary artery disease; COPD = chronic obstructive pulmonary disease; CABG = coronary artery bypass grafting; PCI = percutaneous coronary intervention; NYHA = New York Heart Association; BG = blood glucose; GFR = glomerular filtration rate; BNP  =  brain natriuretic peptide; EF  =  ejection fraction; LVEDD  =  left ventricular end-diastolic diameter; MR = mitral regurgitation; 6-MWT  =  6-minute walk test; MLHF Q =  Minnesota Living With Heart Failure Questionnaire.

### Primary endpoint – safety

The primary endpoint of this study was related to safety with regard to successful clip implantation, in-hospital complication rate, and 30-day mortality. MitraClips have been implanted successfully in all patients with diabetes, whereas one patient in the non-diabetic group had to undergo surgery for significant mitral stenosis. Complications were defined as MACCE (non-diabetes: 5.1% vs. diabetes: 0.0%), sepsis (non-diabetes: 5.1% vs. diabetes: 0.0%), ventilation >24 hours (non-diabetes: 2.6% vs. diabetes: 0.0%), peripheral vascular complications (non-diabetes: 5.1% vs. diabetes: 5.3%), acute kidney injury stage III (0.0% vs. 0.0%), pacemaker damage (non-diabetes: 2.6% vs. diabetes: 0.0%), stroke (0.0% vs. 0.0%) and major bleeding (non-diabetes: 2.6% vs. diabetes: 0.0%). Thirty-day mortality was 2.6% (n = 1) in non-diabetic patients due to a septic event. No patient died in the diabetes group. At the time of follow up overall survival was 98.3% (n = 57) with no death after discharge from hospital in both groups (Table 2).

**Table 2:** Periprocedural results of patients undergoing MitraClip grouped according to the presence of diabetes mellitus type II.

	diabetes (n = 19)	no diabetes (n = 39)	p-value
Successful clip implantation, n (%)	19 (100.0)	38 (97.4)	0.672
multiple clip implantation (>2), n (%)	0 (0.0)	0 (0.0)	
Procedure duration, min ± SD	121±43	120±37	0.895
Radiation time, min ± SD	30±12	27±11	0.388
Significant mitral stenosis, n (%)	0 (0.0)	1 (2.6)	0.672
Peripher vascular complication, n (%)	1 (5.3)	2 (5.1)	0.704
Stroke, n (%)	0 (0.0)	0 (0.0)	
MACCE, n (%)	0 (0.0)	2 (5.1)	0.448
Pacemaker damage, n (%)	0 (0.0)	1 (2.6)	0.672
Sepsis, n (%)	0 (0.0)	2 (5.1)	0.448
Ventilation>24 h, n (%)	0 (0.0)	1 (2.6)	0.672
Acute kidney injury stage III, n (%)	0 (0.0)	0 (0.0)	
Major bleeding, n (%)	0 (0.0)	1 (2.6)	0.672
Mitral valve surgery, n (%)	0 (0.0)	1 (2.6)	0.672
Death			
periprocedural (<72 hours), n (%)	0 (0.0)	0 (0.0)	
30-day mortality, n (%)	0 (0.0)	1 (2.6)	0.672

MACCE = major cardiac and cerebrovascular events.

Major bleeding was defined according to VARC II as overt bleeding either associated with a drop in hemoglobin level of at least 3.0 g/dL or requiring transfusion of two or three units of whole blood, or causing hospitalization, or permanent injury, or requiring surgery and does not meet criteria of life-threatening or disabling bleeding.

### Secondary endpoint – efficacy

The secondary endpoints were related to efficacy with regard to procedure duration (non-diabetes: 120±37 min vs. diabetes: 121±43 min; p >0.05), multiple clip implantation (0.0% vs. 0.0%) and radiation time (non-diabetes: 27±11 min vs. diabetes: 30±12 min; p >0.05).

**Figure 2:**
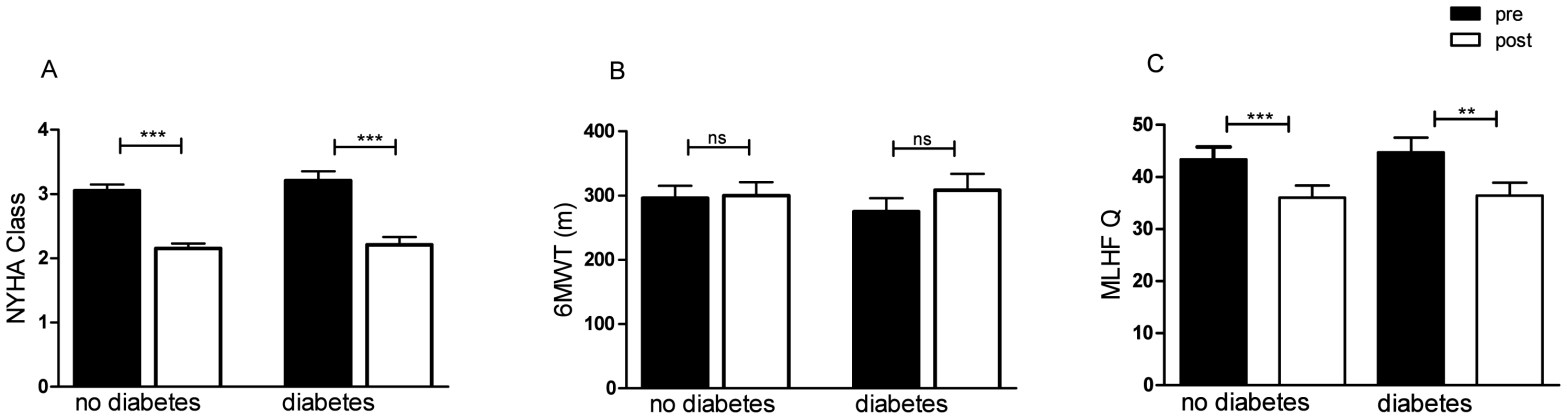
Changes in functional parameters after short-term follow up (3 months). **A** NYHA class before and 3 months after MitraClip. **B** 6-MWT before and 3 months after MitraClip. **C** Results of MLHF Q before and 3 months after MitraClip. NYHA = New York Heart Association; 6-MWT = 6-minute walk test; MLHF Q = Minnesota Living With Heart Failure Questionnaire. ns =  non significant (p≥0.05). *** denotes p<0.0001, ** denotes p<0.001.

**Figure 3:**
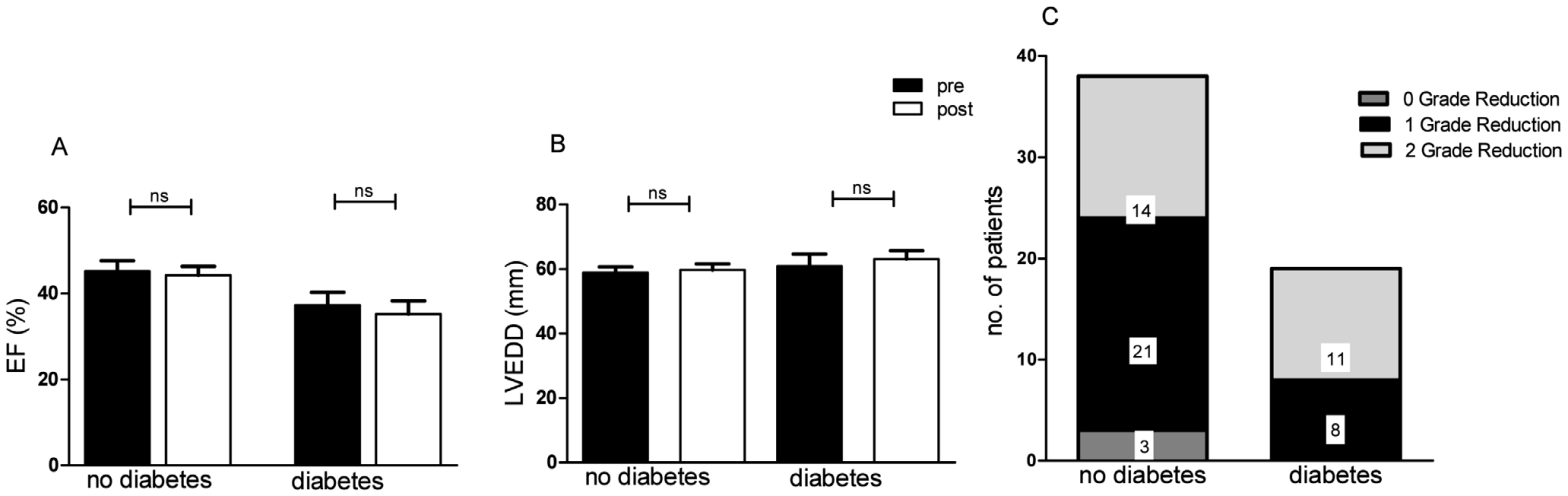
Changes in cardiac parameters after short-term follow up (3 month) in patients undergoing MitraClip. **A** Ejection fraction before and 3 months after MitraClip. **B** Left ventricular end-diastolic diameter before and 3 months after MitraClip. **C** Grade of reduction of mitral regurgitation 3 months after MitraClip. EF = ejection fraction; LVEDD = left ventricular end-diastolic diameter. ns =  non significant (p≥0.05).

At the time of follow up, significant improvement of NYHA class was seen in both groups from 3.1±0.6 to 2.2±0.5 (p<0.0001) in patients without diabetes and 3.2±0.6 to 2.2±0.5 (p<0.0001) in patients with diabetes. Quality of life measured by the MLHF Questionnaire was improved with a score reduction from 43±15 to 36±14 (p<0.0001) in non-diabetic patients and 45±12 to 36±11 (p<0.001) in patients with diabetes (Figure 2). 6-MWT (296±121.6 m to 300±130.8 m; p = 0.743), left ventricular ejection fraction (45±16% to 44±13%; p = 0.660), and left ventricular end-diastolic diameter (59±11 mm to 60±12 mm; p = 0.163) did not change in patients without diabetes. In patients with diabetes 6-MWT (275±90 m to 308±11 m; p = 0.057), ejection fraction (37±13% to 35±13%; p = 0.455) and left ventricular end-diastolic diameter (61±14 mm to 63±11 mm; p = 0.243) were also unaffected. MR reduction ≥1 grade was observed in 65.8% (n = 25) of patients without diabetes and in all patients with diabetes (Figure 3).

## Discussion

The key findings of our study are: i) mitral valve repair with the MitraClip system is safe and effective in patients with diabetes mellitus. ii) This is associated with an improvement in functional status after three months follow up.

Patients with diabetes are at higher risk when undergoing PCI or cardiac surgery [8]–[15]. The underlying mechanisms are multifarious and involve an increased platelet activity, endothelial dysfunction [22], [23], impaired wound healing, and a higher risk for infections caused by hyperglycemia [24], [25]. Patients with diabetes moreover often present with comorbidities such as impaired renal function or coronary artery disease, which increase the surgical risk. PMVR with the MitraClip system has been shown to be safe and feasible and an alternative for surgical mitral valve repair in high-risk patients [7], [20]. The role of diabetes in those patients, however, has not been investigated so far.

In our study population none of the diabetic patients died within 3 months follow-up. However, slightly higher mortality rates have been described [26]–[28]. These high mortality rates may reflect the morbidity of the patients who are referred to MitraClip procedure - sometimes as palliative therapy - and the importance of careful patient selection and risk stratification. Successful clip implantation was 100% in the diabetic group and 97.4% in the non-diabetic group. Overall complication rate was low in our study population, which has also been proved in other studies [29], [30] and confirms the safety of the procedure. Diabetic patients did not present with a higher complication rate.

Previous studies have identified diabetes mellitus to be a risk factor for early and late mortality after surgical mitral valve repair [31], [32], [35]. Therefore it has been included in risk models like the Euroscore II or the STS score. Hospital mortality rates for mitral valve repair have been described from 1.5% to 6.5% [36], [37]. Complications like deep sternal wound infection, stroke or acute renal failure after cardiac surgery are more likely in diabetic patients [33], [34], [35]. A less invasive treatment option like the MitraClip procedure in diabetic patients therefore seems to be an good alternative. Clinical outcome was improved after three months follow up in patients with diabetes and in non-diabetic patients. NYHA class was reduced and quality of life improved. These findings consist with other reports [26], [28]. No improvement was observed in the 6-MWT in both groups, which may be due to the short-term follow up of three months in our study. In previous studies 6MWT was improved after 12 months [26], [29]. Echocardiographic evaluation after three months showed a good result with an MR reduction ≥1 grade in 93.1% (n = 54) of the patients. All patients in the diabetes group did benefit from PMVR with an MR reduction ≥1 grade. There were no relevant changes in ejection fraction and LVEDD in our study population, which may be due to the short term period of follow up. Baseline ejection fraction was moderately to severely reduced in both groups and left ventricular end-diastolic diameter enlarged. Recovery might be seen after a longer period. In contrast, a previously published study reported a reduction of left ventricular end-diastolic volume after short-term follow up of 30 days and one year probably due to early reverse remodeling [28].

Our study has limitations. PMVR using the MitraClip system is safe and event rates are low. Therefore, a prospective randomized study with more patients and longer follow-up time is needed. Furthermore, patients should be classified concerning the duration of diabetes and diabetes-induced comorbidities.

## Conclusion

We here show that the MitraClip procedure can be performed safely and effectively in patients with type II diabetes mellitus. Our short-term follow up shows an improvement in functional status in our patients with no negative influence on 3-month-mortality.

## Supporting Information

Checklist S1 **CONSORT Checklist.**
(DOC)

Protocol S1 **Trial Protocol.**
(DOC)
