# Supplementary material for: Safety and Efficacy of Percutaneous Mitral Valve Repair Using the MitraClip® System in Patients with Diabetes Mellitus
Source: PLoS One. 2014 Nov 6;9(11):e111178. doi: 10.1371/journal.pone.0111178 (PMC4222883; doi:10.1371/journal.pone.0111178)
Supplement: Protocol S1 — Trial Protocol. (DOC) [file pone.0111178.s002.doc]

**Klinik für Kardiologie, Pneumologie und Angiologie**

**Direktor der Klinik**

Univ.-Prof. Dr. med. M. Kelm

Clinical Study Outline

Safety and efficacy of percutaneous mitral valve repair using the MitraClip® system in patients with diabetes mellitus – The MitraClip® Registry

Version 1.0

| **Principal Investigator** | Univ.- Prof. Tienush Rassaf, MD  Klinik für Kardiologie, Pneumologie und Angiologie Moorenstr. 5 40225 Düsseldorf |
| --- | --- |
| **Sub-Investigators** | Jan Balzer, MD  Silke van Hall, MD  Katharina Hellhammer, MD  Christos Rammos, MD  Tobias Zeus, MD |
| **Participating Scientists** | Rabea Wagstaff, B.Sc., M.A. |

***Confidential***

# Abbreviations

| 6-MWT | 6-Minutes-Walk-Test |
| --- | --- |
| GCP | Good Clinical Practice |
| MLHF Q | Minnesota Living With Heart Failure Questionnaire |
| MR | mitral regurgitation |
| PMVR | Percutaneous mitral valve repair |
| QoL | Quality of life |
| TEE | Trans-esophageal echocardiography |
| TTE | Trans-thoracic echocardiography |

| **Title of Study** | **Safety and efficacy of percutaneous mitral valve repair using the MitraClip® system in patients with diabetes mellitus – The MitraClip® Registry** |
| --- | --- |
| **Principal Investigator** | Univ.- Prof. Tienush Rassaf, MD |
| **Sub-Investigators** | Jan Balzer, MD  Silke van Hall, MD  Katharina Hellhammer, MD  Christos Rammos, MD  Tobias Zeus, MD |
| **Participating Scientists** | Rabea Wagstaff, B.Sc., M.A. |
| **Study Duration** | First patient in: Q1 2013  Recruitment Phase: 12 months Observational period per patient: 3 months  Last patient out: Q2 2014 |
| **Legal Aspects** | The study will be performed in accordance with the Declaration of Helsinki and the Good clinical practice (GCP)-guideline. |
| **Background** | Mitral valve regurgitation (MR) is the second common valvular heart disease. Percutaneous mitral valve repair (PMVR) with the MitraClip® system is an effective therapeutic alternative for patients who cannot undergo surgery due to high surgical risk. Patients with diabetes mellitus show a negative outcome in percutaneous coronary intervention, aortic valve replacement and cardiac surgery. The impact of diabetes on treatment of MR using the MitraClip® system is not known. Therefore the aim of this observational study is to assess percutaneous mitral valve repair with the MitraClip® system is safe and effective in patients with diabetes mellitus. |
| **Risk-Benefit-Assessment** | All patients will be discussed in the institutional heart team and declined for surgical treatment due to high operative risk. Percutaneous mitral valve repair (PMVR) with the MitraClip® system is an effective therapeutic alternative for patients who cannot undergo surgery due to high surgical risk. Only patient with symptomatic severe or moderate-to-severe MR referred to PMVR with the MitraClip® system will be included in this observational study. |
| **Objectives** | The aim of the study is assess the safety and efficacy of percutaneous mitral valve repair with the MitraClip® system in patients with diabetes mellitus.  **Primary outcome:** The primary endpoint is related to safety with regard to successful MitraClip® implantation, in-hospital complication rate, and 30-day mortality.  **Secondary outcomes:** The secondary endpoints are related to efficacy with regard to procedure duration and radiation time. Further secondary endpoints are changes in MR and clinical parameters: NYHA class, Quality of life, 6-Minutes-Walk-Test (6-MWT), left ventricular ejection fraction, left ventricular end-diastolic diameter. |
| **Study Design** | non-interventional, open-label, mono-center, observational study |
| **Inclusion Criteria** | - Male and female patients aged > 18 years - Patients with symptomatic severe or moderate-to-severe MR undergo PMVR with the MitraClip® system - Group 1: patients with type II diabetes mellitus (oral medication or on insulin therapy) - Group 2: non-diabetic patients - Written informed consent |
| **Exclusion Criteria** | - Patients unwilling or unable to give informed consent - Underage persons |
| **Number of Patients** | All patients with symptomatic severe or moderate-to-severe MR with or without diabetes mellitus type II who will undergo a PMVR with the MitraClip® System at the Heart Center Duesseldorf will be asked to participate in this observational study. The recruitment period will be 12 months. |
| **Intervention** | All included patients with symptomatic severe or moderate-to-severe MR evaluated by trans-thoracic and trans-esophageal echocardiography will undergo PMVR with the MitraClip® System at the Heart Center Duesseldorf according to local standard. The MitraClip® procedure will be performed either in general anesthesia or deep sedation using TEE and fluoroscopy for guidance. |
| **Methods** | After patients given their written informed consent the following data will be collected:   - Demographics and medical history - physical examination and vital signs - medical imaging - periprocedural in hospital data, e.g. procedure duration and radiation time - laboratory parameters (blood samples for biochemistry and hematology analysis) - Trans-thoracic echocardiography (TTE) - Trans-esophageal echocardiography (TEE) - quality of life measured by the Minnesota Living With Heart Failure Questionnaire (MLHF Q)   One follow-up visit will be performed routinely after 3 months. The following outcome parameters will be collected:   - physical examination and vital signs - medical history - NYHA classification - 6-MWT - quality of life measured by the Minnesota Living With Heart Failure Questionnaire (MLHF Q) - Trans-thoracic echocardiography (TTE) - laboratory parameters (blood samples for biochemistry and hematology analysis) |
| **Data Documentation** | All data to be collected will directly be entered into a suitable data base. |
| **Sample Size** | No sample size calculation will be performed because this is an observational pilot study. |
| **Analysis and Statistics** | Continuous data will be expressed as mean ± standard deviation (SD) and compared with the unpaired student`s t-test or Mann-Whitney U test if not normally distributed. Normality will be checked with the Kolmogorov-Smirnov test. Categorical variables will be evaluated as percentage and compared with the qui-square test or Fisher`s exact test. Statistical analysis will be performed with SPSS® Statistics 22 (IBM®) and Prism® (GraphPad®). A p-value <0.05 is considered to be significant. |
| **Ethics and Quality Assurance** | The study will be approved by the local ethics committee. The study will be conducted according to the ICH-GCP criteria of ”Good Clinical Practice“. It will be performed according to the legal data protection requirements. The pseudonymized data will be archived for 5 years at the University Hospital Duesseldorf. Data analysis will be performed pseudonymized. Data protocol and publication will be anonymized. The patients have the right to receive information. |
| **Insurance** | No patients’ insurance is necessary because this is an observational trial. The patients will be treated within the standard therapy for symptomatic severe or moderate to-severe MR. |

**Literature**

1. Iung B, Baron G, Butchart EG, Delahaye F, Gohlke-Bärwolf C, et al. A prospective survey of patients with valvular heart disease in Europe: The Euro Heart Survey on Valvular Heart Disease. Eur Heart J 2003; 24:1231-43.

2. Nkomo VT, Gardin JM, Skelton TN, Gottdiener JS, Scott CG, et al. The burden of valvular heart diseases: a population-based study. Lancet 2006; 368(9540):1005-11.

3. Rossi A, Dini FL, Faggiano P, Agricola E, Cicoira M, et al. Independent prognostic value of functional mitral regurgitation in patients with heart failure. A quantitative analysis of 1256 patients with ischaemic and non-ischaemic dilated cardiomyopathy. Heart 2011; 97(20):1675-80.

4. Trichon BH, Felker GM, Shaw LK, Cabell CH, O'Connor CM. Relation of frequency and severity of mitral regurgitation to survival among patients with left ventricular systolic dysfunction and heart failure. Am J Cardiol 2003 March 1;91(5):538-43.

5. Patel JB, Borgeson DD, Barnes ME, Rihal CS, Daly RC, et al. Mitral regurgitation in patients with advanced systolic heart failure. J Card Fail 2004; 10(4):285-91.

6. Bonow RO, Carabello BA, Chatterjee K, de Leon AC Jr, Faxon DP, et al. 2008 focused update incorporated into the ACC/AHA 2006 guidelines for the management of patients with valvular heart disease: a report of the American College of Cardiology/American Heart Association Task Force on Practice Guidelines. Journal of the American College of Cardiology 2008; 52 (13):e1-142.

7. Feldman T, Foster E, Glower DD, Kar S, Rinaldi MJ, et al. Percutaneous repair or surgery for mitral regurgitation. N Engl J Med 2011; 364(15):1395-406.

8. Barsness GW, Peterson ED, Ohman EM, Nelson CL, DeLong ER, et al. Relationship between diabetes mellitus and long-term survival after coronary bypass and angioplasty. Circulation 1997; 96:2551– 6.

9. Hillegass WB, Patel MR, Klein LW, Gurm HS, Brennan JM, et al. Long-term outcomes of older diabetic patients after percutaneous coronary stenting in the United States: a report from the National Cardiovascular Data Registry, 2004 to 2008. J Am Coll Cardiol. 2012; 60(22):2280-9.

10. Halkos ME1, Kilgo P, Lattouf OM, Puskas JD, Cooper WA, et al. The effect of diabetes mellitus on in-hospital and long-term outcomes after heart valve operations. Ann Thorac Surg 2010; 90 (1):124–130

11. Feldman T, Kar S, Rinaldi M, Fail P, Hermiller JT, et al. Percutaneous mitral repair with the MitraClip system: safety and midterm durability in the initial EVEREST (Endovascular Valve Edge-to-Edge REpair Study) cohort. J Am Coll Cardiol 2009; 54 (8): 686–694

**Study Flow Chart**

Table 1: Visit Schedule

| **Procedure** | **Screening/Baseline Visit**  **V1** | **Procedure**  **V2** | **Follow-up Visit**  **V3**  **(after 3 month)** |
| --- | --- | --- | --- |
| Informed Consent | X |  |  |
| In-/Exclusion Criteria | X |  |  |
| Physical Examination | X |  | X |
| Medical History | X |  | X |
| Vital Signs | X |  | X |
| NYHA classification | X |  | X |
| 6-MWT | X |  | X |
| QoL | X |  | X |
| TTE | X |  | X |
| TEE | X | X |  |
| periprocedural data (e.g. procedure time, radiation time) |  | X |  |
| Laboratory analysis* | X |  | X |
| Adverse event assessment |  | X | X |

* Nt-pro BNP, GFR, Creatinine, Urea, hs C-reactive Protein, Procalcitonin, blood count, blood glucose, HbA1c

This clinical investigation plan was subject to critical review and has been approved by the following:

**Principal Investigator**

I agree that this Clinical Study Protocol contains all the information required to conduct this study. By my signature below, I hereby certify that I have read, understood and agree to abide by all conditions, instructions and restrictions contained in this clinical investigation plan.

**Principal Investigator**

Date name
